# Computational analysis of heart valve growth and remodeling after the Ross procedure

**DOI:** 10.1007/s10237-024-01874-y

**Published:** 2024-09-13

**Authors:** Elmer Middendorp, Fabian Braeu, Frank P. T. Baaijens, Jay D. Humphrey, Christian J. Cyron, Sandra Loerakker

**Affiliations:** 1 Department of Biomedical Engineering, Eindhoven University of Technology, Eindhoven, The Netherlands; 2 Institute for Complex Molecular Systems, Eindhoven University of Technology, Eindhoven, The Netherlands; 3 Singapore Eye Research Institute, Singapore National Eye Center, Singapore, Singapore; 4 Yong Loo Lin School of Medicine, National University of Singapore, Singapore, Singapore; 5 Singapore-MIT Alliance for Research and Technology, Singapore, Singapore; 6 Department of Biomedical Engineering, Yale University, New Haven, USA; 7 Institute for Continuum and Material Mechanics, Hamburg University of Technology, Hamburg, Germany; 8 Helmholtz-Zentrum, Institute for Material Systems Modeling, Geesthacht, Germany

**Keywords:** Ross procedure, Heart valve, Growth and remodeling, Computational modeling, Mechanobiology

## Abstract

During the Ross procedure, an aortic heart valve is replaced by a patient’s own pulmonary valve. The pulmonary autograft subsequently undergoes substantial growth and remodeling (G&R) due to its exposure to increased hemodynamic loads. In this study, we developed a homogenized constrained mixture model to understand the observed adaptation of the autograft leaflets in response to the changed hemodynamic environment. This model was based on the hypothesis that tissue G&R aims to preserve mechanical homeostasis for each tissue constituent. To model the Ross procedure, we simulated the exposure of a pulmonary valve to aortic pressure conditions and the subsequent G&R of the valve. Specifically, we investigated the effects of assuming either stress- or stretch-based mechanical homeostasis, the use of blood pressure control, and the effect of root dilation. With this model, we could explain different observations from published clinical studies, such as the increase in thickness, change in collagen organization, and change in tissue composition. In addition, we found that G&R based on stress-based homeostasis could better capture the observed changes in tissue composition than G&R based on stretch-based homeostasis, and that root dilation or blood pressure control can result in more leaflet elongation. Finally, our model demonstrated that successful adaptation can only occur when the mechanically induced tissue deposition is sufficiently larger than tissue degradation, such that leaflet thickening overrules leaflet dilation. In conclusion, our findings demonstrated that G&R based on mechanical homeostasis can capture the observed heart valve adaptation after the Ross procedure. Finally, this study presents a novel homogenized mixture model that can be used to investigate other cases of heart valve G&R as well.

**Supplementary Information:**

The online version contains supplementary material available at 10.1007/s10237-024-01874-y.

## Introduction

Heart valve replacements are crucial medical interventions for patients suffering from severe valvular heart disease (Huh and Bakaeen 2006; Hammermeister et al. 2000; Goldstone et al. 2017). Currently, the two primary options for replacing a defective heart valve are bio-prosthetic and mechanical valves (Huh and Bakaeen 2006; Hammermeister et al. 2000; Goldstone et al. 2017). Albeit lifesaving, both types of heart valve prostheses are associated with major drawbacks. For example, bio-prosthetic valves are prone to accelerated degeneration (Arsalan and Walther 2016), rendering them unsuitable for young patients, and mechanical valves require lifelong anticoagulation therapy (Roudaut et al. 2007). In addition, both types of prosthetic valves are non-living structures. Living valve replacements may be able to, at least partly, overcome the limitations of contemporary valve replacements because they can accommodate somatic growth and adapt to changes in the hemodynamic demands of the patient (Fioretta et al. 2021). Promising options for living heart valve replacements are tissue-engineered heart valve (TEHVs) and autografts.

In situ heart valve tissue engineering aims to create living heart valves, with growth and remodeling (G&R) capacity, at the site of destination (Kluin et al. 2017; Wissing et al. 2017). This entails implantation of a bio-instructive scaffold directly at the functional site, which is subsequently populated by various cell types. These cells synthesize extracellular matrix (ECM) proteins and degrade the scaffold material, which is hypothesized to result in the establishment of a new, homeostatic, living valve. However, despite completed (Morales et al. 2021) and ongoing (Xeltis Xplore-2, NCT03022708) clinical trials, TEHVs have not reached clinical practice yet. This means that the use of an autograft is currently the only clinically available option for a living valve replacement.

Autografts can be used according to the Ross procedure, in which a defective aortic valve is replaced by the patient’s own pulmonary valve (Ross 1967; Mazine et al. 2018). Since cells in this pulmonary valve remain alive, the autograft can adapt to its new environment through deposition, degradation and remodeling of ECM proteins. As a consequence, explanted autografts have often been reported to feature a dilated neo-aortic root and increased leaflet thickness, in combination with the addition of a fibro-elastic tissue layer at the ventricular surface of the leaflet (Schoof et al. 2000; Yacoub et al. 2020; Mookhoek et al. 2010; Rabkin-Aikawa et al. 2004). Since root dilation is commonly associated with valve regurgitation, several studies have been performed to better understand and prevent this dilation using (degradable) reinforcement materials (Schoof et al. 1998, 2000; Xie et al. (2001; Kouchoukos et al. 2004; Rabkin-Aikawa et al. 2004; Schoof et al. 2006; Charitos et al. 2009; Luciani et al. 2010; Mookhoek et al. 2010, 2016; Famaey et al. 2018; Vastmans et al. 2018; Van Hoof et al. 2021; Maes et al. 2023; Vervenne et al. 2023). In contrast, the G&R response of the leaflets remains poorly understood. An improved understanding of leaflet G&R after the Ross procedure may help to identify strategies that ensure successful adaptation of the pulmonary valve to the aortic position with increased hemodynamic loading. In addition, these insights may also contribute to improving the outcomes of TEHVs.

The G&R response of cardiovascular tissues relies heavily on ECM turnover by cells, which is modulated by a multitude of (bio-)chemical and mechanical stimuli (Karakaya et al. 2021; Abdulghani and Mitchell 2019; Loon et al. 2013; Ikhumetse et al. 2006). Notwithstanding the important roles of biochemical stimuli, which are extensively reviewed in Abdulghani and Mitchell (2019) and Loon et al. (2013), in this study we aim to investigate the role of the mechanical environment on the G&R of Ross autografts. The effects of mechanical stimuli are complex to investigate via experimental approaches alone because mechanical parameters (e.g., stress, strain, stiffness) vary both in space and in time and are difficult to measure *in vivo*. Computational models can make important contributions to understanding and predicting the interplay between mechanical cues and G&R and to designing future experiments (Loerakker and Humphrey 2022).

For example, using a computational model of heart valve mechanics, we demonstrated that valve geometry has a large impact on the mechanical state of TEHVs and discovered that a frequently used valve geometry could be responsible for the adverse remodeling of TEHVs observed in vivo (Loerakker et al. 2013). We used our computational model to propose a potentially improved valve geometry (Loerakker et al. 2013; Sanders et al. 2016), and a subsequent pre-clinical study incorporating this different geometry confirmed a significantly improved patency of TEHVs due to improved valve remodeling consistent with computational predictions (Emmert et al. 2018). In addition, Motta et al. 2020 showed that this more physiological geometry also resulted in a lower inflammatory response and reduced activation of myofibroblasts. These results demonstrate that mechanical stimuli play a pivotal role during heart valve G&R, and that computational models of mechano-regulated G&R can provide unique insights that can be used to improve the adaptation and long-term functionality of living heart valve replacements.

To describe the adaptation of the pulmonary autograft after the Ross procedure, a computational framework is needed that can describe both the observed changes in tissue mass (i.e., growth), composition, and structure (i.e., remodeling) (Schoof et al. 2000; Yacoub et al. 2020; Mookhoek et al. 2010; Rabkin-Aikawa et al. 2004). Describing the full G&R process is possible with constrained mixture models, in which the continuous production and removal of individual ECM components are evaluated independently (Humphrey and Rajagopal 2002). Yet, a classical constrained mixture model is computationally intractable for heart valve geometries, since numerical valve models require a spatial discretization with many integration points. To overcome this intractability, we adopted a homogenized constrained mixture framework to investigate heart valve G&R. Specifically, we approximated the classical hereditary integral by a temporal homogenization of constituent-specific stress-free state according to Cyron et al. (2016) which results in a computational model that can be efficiently evaluated in a finite element setting.

With this homogenized constrained mixture model, we aimed to investigate the role of mechano-regulated G&R in the adaptation of the autograft after the Ross procedure. We first investigated whether stress- or stretch-based homeostasis, as competing hypotheses, best describes the observed in vivo G&R response. Subsequently, we used our model to analyze how blood pressure control and root dilation may affect the G&R response of the autograft in response to increased hemodynamic loading conditions.

## Methods

To simulate G&R after the Ross procedure, we modeled heart valves as a homogenized constrained mixture of elastin, glycosaminoglycans (GAGs), and collagen fibers. In this model, collagen and GAGs were continuously deposited and degraded, while elastin production and degradation were assumed to be negligible throughout the simulations since adult valves were studied (Votteler et al. 2013). The degradation and deposition of collagens and GAGs were not necessarily equal, such that increases or decreases in the apparent mass density of these constituents could occur, which consequently translated into changes in the composition, stiffness and overall volume of the heart valve. In addition, the newly synthesized collagen fibers and GAGs were assumed to be pre-stretched upon incorporation into the ECM. The deposition stress (that corresponded with its pre-stretch) in each new mass increment of collagen fibers or GAGs (i.e., the mass of collagen or GAGs deposited at a single point in time) could differ from the current stress in the previously deposited mass increments of these constituents. The corresponding changes in the homogenized (i.e., mass-averaged) stress-free state of each constituent were accounted for in the model. Together, these constituent-specific changes in apparent mass density and stress-free state described the overall G&R response of the tissue.

The homogenized constrained mixture framework was implemented in the user subroutine UMAT in Abaqus Standard (Abaqus 2018, Dassault Systèmes Simulia Corp. Johnston, RI, USA). Abaqus implicit was used to solve the equations related to the balance of linear momentum, while explicit time integration was used only to solve the evolution equations regarding the constituent apparent mass densities and homogenized stress-free states (with a maximum timestep of 0.25 days).

### Governing equations

Throughout this paper, we adopted the following notations: Vectors are indicated with a bold lowercase symbol ($\textbf{e}$) and second-order tensors are shown in bold uppercase ($\textbf{F}$). In Einstein notation, the inner product was defined as $\textbf{A}\cdot \textbf{B}=A_{ik}B_{kj} e_i e_j$, the double contraction as $\textbf{A}:\textbf{B}=A_{ij}B_{ij}$, and the dyadic product as $\textbf{A}\otimes \textbf{B}=A_{ij}B_{kl}e_i e_j e_k e_l$.

With these definitions, consider a body with a reference configuration $\Omega _0$ that deforms to configuration $\Omega _s$. A line segment $d\textbf{x}_0$ in $\Omega _0$ can then be mapped to its deformed equivalent $d\textbf{x}_s$ in $\Omega _s$ using the deformation gradient tensor $\textbf{F}$:

1 $$d\textbf{x}_s=\textbf{F}\cdot d\textbf{x}_0.$$

Using this, a displacement field ($\textbf{u}=d\textbf{x}_s-d\textbf{x}_0$) was determined that corresponded with a stress field (with $\varvec{\sigma}$ the Cauchy stress tensor) that satisfied the balance of linear momentum in which inertial and body forces are assumed negligible:

2 $$\nabla \cdot \varvec{\sigma} = \textbf{0}.$$

The homogenized constrained mixture approach entailed a split of the deformation gradient tensor into an elastic and an inelastic part for each individual constituent (Cyron et al. 2016):

3 $$\begin{aligned} \textbf{F}=\textbf{F}_e^{i}\cdot \textbf{F}_r^{i}. \end{aligned}$$

Here, $\textbf{F}^i_e$ described the homogenized elastic deformation of all mass increments belonging to constituent *i*, and $\textbf{F}_r^i$ described the deformation from the reference configuration to the homogenized, constituent-specific, stress-free state. From the elastic deformation gradient tensor, the constituent-specific elastic right Cauchy–Green tensor was obtained and used to define the mechanical behavior of a constituent:

4 $$\begin{aligned} \textbf{C}_e^i=\textbf{F}_e^{i,T}\cdot \textbf{F}_e^{i}. \end{aligned}$$

The strain energy (per unit of mass) of constituent *i* ($W^i$) was defined as a function of $\textbf{C}_e^i$, which allowed the construction of the constituent-level second Piola–Kirchhoff stress ($\textbf{S}^i$) according to:

5 $$\begin{aligned} \textbf{S}^i=\frac{2\rho _R^i}{\varphi ^i}\frac{\partial W^i}{\partial \textbf{C}} . \end{aligned}$$

In this equation, $\rho _R^i$ represented the apparent mass density of constituent *i* (which is defined in the reference configuration) and $\varphi ^i$ indicates its volume-fraction (in the current configuration). Using the standard push-forward operation with $J=\det \textbf{F}$, the constituent-level Cauchy stress $\varvec{\sigma} ^i$ was obtained:

6 $$\begin{aligned} \varvec{\sigma} ^i=\frac{1}{J}\textbf{F}\cdot \textbf{S}^i\cdot \textbf{F}^{T}. \end{aligned}$$

Finally, the Cauchy stress of the complete tissue was determined by summing all constituent-level stresses and a penalty stress $\varvec{\sigma} ^*$:

7 $$\begin{aligned} \varvec{\sigma} =\sum ^{n_\text{const}}_{i=1}\varphi ^i\varvec{\sigma} ^i+\varvec{\sigma} ^*. \end{aligned}$$

The penalty stress aimed to ensure that the change in the total mass of the tissue led to an equivalent change in tissue volume (i.e., assuming a constant spatial density of the tissue). The specific definition of this penalty stress is provided in Sect. 2.2.4.

Cyron et al. (2016) showed that the evolution of the stress-free configuration (mapped with $\textbf{F}_r^i$) can be determined from:

8 $$\begin{aligned} & \left[\frac{\dot{\rho _R^i}}{\rho _R^i}+\frac{1}{T^i}\right][\textbf{S}^i-\textbf{S}^i_{dep}] \\ & \quad= \left[2\frac{\partial \textbf{S}^i}{\partial \textbf{C}_e^i}:(\textbf{C}_e^i\cdot \dot{\textbf{F}_r^i}\cdot \textbf{F}_{r}^{i,-1})\right]_{\textbf{F}=const}. \end{aligned}$$

Here, the overdot indicated a time derivative, $T^i$ was the average turnover time of constituent *i*, and $\textbf{S}_{dep}^i$ was defined as the constituent-specific, deposition second Piola–Kirchhoff stress (i.e., the second Piola–Kirchhoff stress of the newly deposited constituent at its pre-stretch):

9 $$\begin{aligned} \textbf{S}_{dep}^i=\textbf{S}(\textbf{F}^i_e=\textbf{F}^i_{e,prestretch}). \end{aligned}$$

By substituting the constituent-specific constitutive laws for the current and depositional second Piola–Kirchhoff stress ($\textbf{S}^i$ and $\textbf{S}^i_{dep}$) and its derivative ($\partial {\textbf{S}^i}/\partial {\textbf{C}_e^i}$) in equation 8, this equation could be re-arranged into a constituent-specific differential equation that allowed the computation of $\textbf{F}_r^i$.

The evolution of the apparent density of constituent *i* ($\rho _R^i$) depended on a stimulus function $\eta ^i$ with corresponding gain parameter $k_{\eta }^i$:

10 $$\begin{aligned} \dot{\rho _R^i}=\rho _R^i k_\eta ^i\eta ^i. \end{aligned}$$

The stimulus function described how tissue deposition was regulated by mechanical factors, often with respect to a certain homeostatic target value. Specifically, when the stimulus function equaled zero, production and removal of constituent *i* were balanced; when the function was positive, more mass of constituent *i* was produced than degraded; when its value was negative, degradation of constituent *i* outweighed its production.

### Constituent-specific constitutive equations

#### Elastin

We modeled the mechanical contribution of elastin using a neo-Hookean strain energy function:

11 $$\begin{aligned} W^{el}=\frac{\mu ^{el}}{2}(I_{1,e}^{el}-3-2\ln (J_{el}^e)), \end{aligned}$$

where $\mu ^{el}$ indicated the shear modulus, $I_{1,e}^{el}$ was the first invariant of the elastin-specific, elastic, right Cauchy–Green tensor $\textbf{C}_e^{el}$ ($I_{1,e}^{el}=\textbf{C}_e^{el}:\textbf{I}$) and $J_e^{el}$ was the square root of its third invariant ($J_e^{el}=\sqrt{\det (\textbf{C}_e^{el})}$). This strain energy function for elastin led to the following second Piola–Kirchhoff stress:

12 $$\begin{aligned} \textbf{S}^{el}=\frac{\mu ^{el}\rho _R^{el}}{\varphi ^{el}}(\textbf{C}_{r}^{el,-1}-\textbf{C}^{-1}) . \end{aligned}$$

This stress was a function of the inverse of the elastin-specific, remodeling right Cauchy–Green tensor ($\textbf{C}_r^{el,-1}=\textbf{F}_r^{el,-1}\cdot \textbf{F}_r^{el,-T}$) and the inverse of the total right Cauchy–Green tensor ($\textbf{C}^{-1}=\textbf{F}^{-1}\cdot \textbf{F}^{-T}$). Performing the push-forward operation described in equation 6 on the elastin-specific second Piola–Kirchhoff stress led to the elastin-specific Cauchy stress:

13 $$\begin{aligned} \varvec{\sigma} ^{el}=\frac{\mu ^{el}}{J}\frac{\rho _R^{el}}{\varphi ^{el}}(\textbf{B}_e^{el}-\textbf{I}) . \end{aligned}$$

Here, $\textbf{I}$ indicated the second order unit tensor and $\textbf{B}_e^{el}=\textbf{F}_e^{el}\cdot \textbf{F}^{el,T}_e$ was the elastin-specific, elastic left Cauchy–Green tensor.

Due to the assumption of negligible elastin turnover, there was no need to define the evolution equations for the apparent mass density or stress-free state of this constituent.

#### Collagen

To model the collagen fibers, we defined 30 fiber directions within the circumferential-radial plane of the leaflet at $6^\circ$ angles. Initial anisotropy in the collagen network was introduced by assigning direction-dependent volume fractions of collagen fibers (adjusted from Oomen et al. (2016)):

14 $$\begin{aligned} \varphi _0^{c,i}&= \frac{1}{A}\left( \exp {\frac{\cos (2(\gamma ^i-\alpha ))+1}{\beta }} \right. \\ &+ \quad \left. \exp {\frac{\cos (2(\gamma ^i-\alpha ))+1}{\beta }}\right) , \end{aligned}$$

15 $$\begin{aligned} A&= \frac{1}{(1-\varphi ^e_0-\varphi ^g_0)} \\ & \quad \sum _{i=1}^{30}\left( \exp {\frac{\cos (2(\gamma ^i-\alpha ))+1}{\beta }}+ \right. \\ & \quad \left. \exp {\frac{\cos (2(\gamma ^i-\alpha ))+1}{\beta }}\right) . \end{aligned}$$

The initial collagen volume fraction in direction $\gamma ^i$ depended on the main fiber angle ($\alpha$) and the dispersity of the fiber network ($\beta$). Finally, the normalization parameter *A* ensured that the total sum of volume fractions remained equal to one.

The mechanical behavior of the collagen fibers was described using a strain stiffening constitutive law (adjusted from Oomen et al. (2016)):

16 $$\begin{aligned} W^{c,i}=\frac{k_1^c}{2k_2^c} \Big (e^{k_2^c\langle (\lambda _e^{c,i})^2-1\rangle }-k_2^c\langle (\lambda _e^{c,i})^2-1\rangle -1). \end{aligned}$$

Here, the elastic fiber stretch was defined as $\lambda _e^{c,i}=\sqrt{\textbf{C}_e^{c,i}:[\textbf{e}_r^{c,i}\otimes \textbf{e}_r^{c,i}}]$ with directional vector $\textbf{e}_r^{c,i}$ indicating the orientation of fiber *i* in its stress-free state. Next, the two parameters $k_1^c$ and $k_2^c$ together determined the linear and nonlinear stiffness of the fiber. Finally, the Macaulay brackets ($\langle \rangle$) ensured that the fibers only contributed to the tissue’s mechanical behavior in extension (i.e., when $\lambda _e^{c,i}>1$).

Based on this strain energy and equation 5 and 6, the collagen-specific second Piola–Kirchhoff stress ($\textbf{S}^{c,i}$) and Cauchy stress ($\varvec{\sigma} ^{c,i}$) in extension were equal to:

17 $$\begin{aligned} \textbf{S}^{c,i}& = \frac{\rho _R^{c,i}}{\varphi ^{c,i}}k_1^c\Big (e^{k_2^c\langle (\lambda _e^{c,i})^2-1\rangle }-1\Big )\frac{1}{(\lambda _r^{c,i})^2}[\textbf{e}_0^{c,i}\otimes \textbf{e}_0^{c,i}], \end{aligned}$$

18 $$\begin{aligned} \varvec{\sigma} ^{c,i}& = \frac{\rho _R^{c,i}}{J\varphi ^{c,i}}k_1^c\Big (e^{k_2^c\langle (\lambda _e^{c,i})^2-1\rangle }-1\Big )(\lambda _e^{c,i})^2[\textbf{e}_s^{c,i}\otimes \textbf{e}_s^{c,i}]. \end{aligned}$$

In these equations, $\lambda _r^{c,i}$ described the homogenized stress-free deformation of the fibers oriented in direction *i*. The directional vectors $\textbf{e}_0^{c,i}$ and $\textbf{e}_s^{c,i}$ defined the direction of fiber *i* in the referential and the deformed configuration, respectively.

To model the G&R behavior of collagen fibers, we adopted direction-dependent stimulus functions for either stretch- and stress-based homeostasis:

19 $$\begin{aligned} \eta ^{c,i}& = \frac{\lambda _e^{c,i}-\lambda _{e,hom}^{c,i}}{\lambda _{e,hom}^{c,i}}, \end{aligned}$$

20 $$\begin{aligned} \eta ^{c,i}& = \frac{\sigma ^{c,i}-\sigma _{hom}^{c,i}}{\sigma _{hom}^{c,i}}. \end{aligned}$$

Specifically, this stimulus depends on either the difference between the current and homeostatic stretch of the collagen fiber under the assumption of stretch-based homeostasis (equation 19) or on the difference between the current scalar Cauchy stress of the collagen fiber and the assumed homeostatic stress in case of stress homeostasis (equation 20). The hypothesis of stretch-based homeostasis was motivated by findings that tissue stretch is similar in semilunar valves over a wide range of ages (Oomen et al. 2016) and that valvular interstitial cells apply a G&R mechanism that aims to restore a normal deformation pattern (Rego et al. 2016). The hypothesis of stress-based homeostasis was motivated by the notion that valvular interstitial cells deposit fibers to restore homeostatic fiber stresses (Rego et al 2016).

By substituting equation 19 and 20 into equation 10, the evolution of the apparent mass-density of the collagen fibers was described with equation 21 or 22 for either stretch- or stress-based homeostasis.

21 $$\begin{aligned} \dot{\rho _R^i}& = \rho _R^ik_{\lambda }^c\frac{\lambda _e^{c,i}-\lambda _{e,hom}^{c,i}}{\lambda _{e,hom}^{c,i}} , \end{aligned}$$

22 $$\begin{aligned} \dot{\rho _R^i}& = \rho _R^ik_{\sigma }^c\frac{\sigma ^{c,i}-\sigma _{hom}^{c,i}}{\sigma _{hom}^{c,i}} . \end{aligned}$$

These definitions finally allowed to re-arrange equation 8 to obtain the evolution equation for the shift in the homogenized stress-free state using:

23 $$\begin{aligned} \dot{\lambda _r^{c,i}}=\frac{\lambda _r^{c,i}}{(\lambda _e^{c,i})^2} \frac{ \Big [\frac{\dot{\rho _R^{c,i}}}{\rho _R^{c,i}}+\frac{1}{T^c}\Big ](S^{c,i}-S^{c,i}_{dep}) }{2\frac{\partial S^{c,i}}{\partial (\lambda _e^{c,i})^2}}. \end{aligned}$$

From this equation, the collagen fiber-specific stress-free state $\lambda _r^{c,i}$ was computed for each point in time using a forward Euler integrative scheme.

#### GAGs

In comparison with the other ECM-proteins, we assumed that GAGs had a relatively low contribution to the mechanical stress in heart valves, but the apparent mass density of GAGs did influence the volume and composition of the tissue. To account for this low contribution to the overall tissue stress, GAGs were assumed to behave according to a neo-Hookean constitutive law with a shear modulus ($$\mu ^g$$) set to 0.1% of the shear modulus of elastin ($$\mu ^e$$). Specifically, the GAGs’ strain energy function ($$W^g)$$, second Piola–Kirchhoff stress ($$\textbf{S}^g$$), and Cauchy stress ($$\varvec{\sigma} ^g$$) equaled:

24 $$\begin{aligned} W^g& = \frac{\mu ^g}{2}(I^g_{1,e}-3-2\ln (J_e^g)), \end{aligned}$$

25 $$\begin{aligned} \textbf{S}^g& = \frac{\mu _g\rho _R^g}{\varphi ^g}(\textbf{C}_r^{g,-1}-\textbf{C}^{-1}), \end{aligned}$$

26 $$\begin{aligned} \varvec{\sigma} ^g& = \frac{\mu ^g}{J}\frac{\rho _R^g}{\varphi ^g}(\textbf{B}_e^g-\textbf{I}). \end{aligned}$$

The GAG stimulus functions for stretch- and stress-based homeostasis were defined based on the first invariants of the GAG-specific left elastic Cauchy–Green tensor and the GAG-specific Cauchy stress, respectively:

27 $$\begin{aligned} \eta ^g& = \frac{(\textbf{B}_e^g-\textbf{B}^g_{e,hom}):\textbf{I}}{\textbf{B}^g_{e,hom}:\textbf{I}}, \end{aligned}$$

28 $$\begin{aligned} \eta ^g& = \frac{(\varvec{\sigma} ^g-\varvec{\sigma} _{hom}^g):\textbf{I}}{\varvec{\sigma} _{hom}^g:\textbf{I}}. \end{aligned}$$

By introducing equation 27 or 28 into equation 10, the evolution of the GAGs’ apparent density was described for a stretch- or stress-based homeostasis as:

29 $$\begin{aligned} \dot{\rho _R^g}& = \rho _R^gk^g_{\lambda }\frac{(\textbf{B}_e^g-\textbf{B}^g_{e,hom}):\textbf{I}}{\textbf{B}^g_{e,hom}:\textbf{I}} , \end{aligned}$$

30 $$\begin{aligned} \dot{\rho _R^g}& = \rho _R^gk^g_{\lambda }\frac{(\varvec{\sigma} ^g-\varvec{\sigma} _\text{hom}^g):\textbf{I}}{\varvec{\sigma} _\text{hom}^g:\textbf{I}} . \end{aligned}$$

With these definitions, the evolution of the homogenized stress-free configuration of GAGs could be determined from equation 8 as (see Appendix A. for derivation):

31 $$\begin{aligned} \dot{\textbf{F}_r^g}=\frac{\varphi ^g}{2\mu ^g\rho _R^g} \big [\frac{\dot{\rho _R^g}}{\rho _R^g}+\frac{1}{T^g}\big ] \textbf{F}_r^g\cdot (\textbf{S}^g-\textbf{S}^g_\text{dep})\cdot \textbf{C} . \end{aligned}$$

From this evolution equation, the homogenized stress-free configuration of GAGs was determined at each timepoint during the G&R response using a forward Euler integrative scheme.

#### Penalty stress

In addition to the ECM proteins that contributed to the overall tissue stress, an additional stress was introduced to preserve a constant mass density of the tissue. As Braeu et al. (2019) demonstrated, such a penalty stress will lead to volumetric growth predominantly in the least stiff direction of the tissue. In this study, the penalty stress was defined as follows:

32 $$\begin{aligned} \varvec{\sigma} ^*=\frac{\kappa }{2J_m}\left(\frac{J}{J_m}-1\right)\textbf{I}, \end{aligned}$$

with

33 $$\begin{aligned} J_m(s)=\frac{\sum _{i=1}^{n_{const}}\rho _R^i(s)}{\rho _0}. \end{aligned}$$

This penalty stress aimed to equalize the volume change of the tissue (*J*) to the change in mass ($J_m$). The change in mass was defined as the summed apparent densities of the tissue at G&R time *s* divided by the tissue density in the reference state. The similarity between *J* and $J_m$ was controlled by the parameter $\kappa$ that acts as a penalty parameter.

### Material property estimation

The three layers of pulmonary heart valve leaflets (i.e., ventricularis, spongiosa, and fibrosa) encompass approximately 20, 35, and 45% of the thickness, respectively (Pierlot et al. 2015a, b; van Rijswijk et al. 2017). Given that these layers are predominantly composed of elastin (ventricularis), GAGs (spongiosa), and collagen (fibrosa) (Ayoub et al. 2016), we set the initial volume fractions of these constituents to 0.2, 0.35, and 0.45, respectively. Under the assumption that the total tissue density equals the density of a well-hydrated tissue (1050 kg/m^3^), the initial apparent densities of these constituents were then calculated from:

34 $$\rho _{R,0}^i=\varphi ^i\rho _0.$$

The material stiffness parameters in our model were derived from measurements of Oomen et al. (2016). In that study, multiple heart-valve leaflets were analyzed using a combination of micro-indentation, digital image correlation, and inverse finite element analysis to determine the in-plane mechanical behavior. Based on the reported material parameters, stress–strain curves for the adult pulmonary leaflets were reconstructed. Using the MATLAB function lsqnonlin (MATLAB R2021a, The MathWorks Inc., Natick, MA, USA), a single set of parameters ($\mu _e$, $k_1^c$, $k_2^c$, and $\beta$) was determined that best approximated the mechanical behavior of all individual valves of Oomen et al. (2016), via minimizing the following residual:

35 $$\begin{aligned} R&= \sum ^{N_{valves}}_{j=1}\left( \sum ^{1000}_{i=1}\left( \frac{[\sigma _j^{circ}(\lambda _i)-\sigma _{HCMM}^{circ}(\lambda _i)]^2}{\sigma _j^{circ}(\lambda _i)}\right.\right. \\ & \quad\quad \left.\left.+\frac{[\sigma _j^{rad}(\lambda _i)-\sigma _{HCMM}^{rad}(\lambda _i)]^2}{\sigma _j^{rad}(\lambda _i)} \right) \right). \end{aligned}$$

In this equation, $\sigma ^{circ}_j$ and $\sigma ^{rad}_j$ are the circumferential and radial stresses of the valves described in Oomen et al. (2016) and $\sigma ^{circ}_{HCMM}$ and $\sigma ^{rad}_{HCMM}$ are the circumferential and radial stresses of the homogenized constrained mixture model. The resulting stress–strain curves are provided in supplementary Fig. 1.

The average lifetime ($T^{i}$) and the parameter that regulates the rate of additional mass deposition out-of-homeostasis ($k_{\lambda}$ and $k_{\sigma}$) for the constituents were also required to describe the G&R response. As stated before, we assumed no turnover of elastin. For collagen and GAGs, an average lifetime of 70 (Famaey et al 2018) and 45 days (Morales and Hascall 1988; Fraser et al. 1997) was adopted. Due to the limited availability of data on increased mass deposition in response to mechanical signals, the gain parameters of collagen and GAGs were assumed to be equal, and a range of values for $k_{\lambda}$ or $k_{\sigma}$ was examined parametrically. Higher gain values were selected for stretch-based homeostasis versus stress-based homeostasis aiming to obtain similar growth and remodeling outcomes despite the clear difference in nonlinearity of the two hypotheses.

Finally, initial values for the remodeling deformation gradient tensors ($\textbf{F}_r^i$

) were required. A collagen deposition stretch of 1.25 and an elastin prestretch of 1.2 in both the circumferential and radial directions were adopted (Famaey et al 2018; Latorre and Humphrey 2018). For simplicity, it was further assumed that the initial GAG prestretch was equal to that of elastin. All parameter values are summarized in Table 1.

**Table 1:** Parameters for the material model

Parameter	Value	Source
$\mu _e$	$119 [\frac{Pa \, m^3}{kg}]$	Fitted on Oomen et al. (2016)
$k_1^c$	$17.0 [\frac{Pa \, m^3}{kg}]$	Fitted on Oomen et al. (2016)
$k_2^c$	$5.25 [-]$	Fitted on Oomen et al. (2016)
$k_3^c$	$25.0 [-]$	Fitted on Oomen et al. (2016)
$\beta$	$0.829 [-]$	Fitted on Oomen et al. (2016)
$\kappa$	0.242[MPa]	Mutlu et al. (2021)
$T^g$	45[days]	Fraser et al. (1997); Morales and Hascall (1988)
$T^c$	70[days]	Famaey et al. (2018)
$k_{\sigma} ^{c}=k_{\sigma} ^{g}$	$\frac{0}{T^i}$, $\frac{0.1}{T^i}$	
	$\frac{0.2}{T^i}$, $\frac{0.3}{T^i} [ days^{-1}]$	[–]
$k_{\lambda} ^c=k_{\lambda} ^g$	$\frac{0}{T^i}$, $\frac{2.0}{T^i}$	
	$\frac{4.0}{T^i}$, $\frac{6.0}{T^i} [days^{-1}]$	[–]
$\lambda _r^c(0)$	$\frac{1}{1.25}$ [-]	Latorre and Humphrey (2018)
$$\lambda ^e_{r,circ}=\lambda ^e_{r,rad}=$$		
$\lambda ^g_{r,circ}=\lambda ^g_{r,rad}$	$\frac{1}{1.2} [-]$	Famaey et al. (2018)

### Simulations

#### Biaxial tensile tests

First, we simulated biaxial tensile tests to gain more mechanistic insights into the mechanisms of G&R in planar tissues with collagen fibers oriented in multiple directions. Initial homeostasis was defined at no deformation beyond the deposition values ($\textbf{F}=\textbf{I}$) and with an initially isotropic collagen fiber organization in the *x*-*y* plane ($\beta =1\cdot 10^4$ [-]). Subsequently, we disturbed this homeostasis by applying a 5% extension in the *x*-direction and either a 5% or a 10% extension in the *y*-direction. Finally, tissue G&R was simulated for 600 days until a new equilibrium was reached. These simulations were performed under the assumption of either stress- or stretch-based homeostasis.

#### Ross procedure

To simulate heart valve G&R after the Ross procedure a Gulbulak valve design (Gulbulak et al. 2020) was defined as the reference geometry for the pulmonary valve. Symmetry was exploited by modeling half a leaflet (1660 linear (C3D8) elements). Nodal displacements were restricted in all directions on the outer edge of the leaflet representing the leaflet’s connection to the pulmonary root. On the symmetry plane of the half-leaflet, nodal displacements were restricted perpendicular to the symmetry plane. Contact between the different leaflets of the valve was simulated using a rigid plate located where the leaflets would normally meet. This contact was assumed to be frictionless and penetrations were negated using the penalty method.

**Fig. 1:**
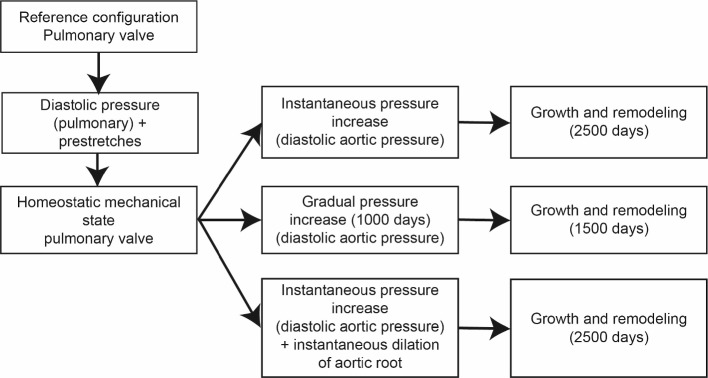
Flowchart describing the simulation procedures for the different scenarios of G&R after the Ross procedure

The Ross procedure was simulated using a two-step procedure (Fig. 1). In the first step, homeostasis was defined under pulmonary conditions (see also section 2.3). Here, we introduced the constituent-specific pre-stretches and applied the normotensive diastolic pulmonary blood pressure of 2 kPa to the outflow surface of the valve. As a consequence of the selection of the constituent-specific pre-stretches, there may be a slight difference between the stress-free, unloaded reference configuration and the initial homeostatic configuration in which the normotensive pulmonary loads and the pre-stretches are accounted for. These differences meant that the homeostatic elastic stretch of the constituents was not exactly equal to the prescribed prestretch, but varied slightly depending on the location in the leaflet. To account for these potential differences, the local homeostatic target stretch (or corresponding stress) was assumed to be equal to the local elastic stretch (or corresponding local stress) in this homeostatic configuration. Finally, a lower bound for the homeostatic target stretch was set at 1.1 (or its corresponding stress) for the collagen fibers to avoid numerical instabilities while evaluating equation 23.

In the second step, the Ross procedure was simulated under three different scenarios, and the subsequent G&R response was analyzed to study the possible re-establishment of homeostasis. In the first scenario, the Ross procedure was simulated by instantaneously increasing the pressure on the leaflet to diastolic systemic values (10 kPa) after which the tissue was allowed to adapt to the altered hemodynamic circumstances for 2500 days. In the second scenario, the effect of the loading rate on the subsequent G&R was studied, by increasing the pressure on the leaflets slowly over 1000 days toward diastolic systemic values. During this period, the tissue was allowed to adapt to the gradual increase in pressure. Subsequently, another 1500 days of growth and remodeling were simulated for which the pressure was kept constant at systemic values. Finally, the third scenario served to study the effect of root dilation on the subsequent G&R response. In this case, in addition to an instantaneous increase in blood pressure to systemic values, the outer diameter of the valve was increased by 20% via imposing nodal displacements at the outer edge in the radial direction. After these changes, 2500 days of G&R were simulated. In all three scenarios, the pressure was reduced back to 2 kPa at the end of the G&R period to evaluate the increase in thickness. The relative change (in percentages) in leaflet thickness was estimated using:

36 $$\begin{aligned} \Delta h=\frac{h_s-h_0}{h_0} \cdot 100 , \end{aligned}$$

with $h_0$ and $h_f$ the leaflet thicknesses before and after G&R, both determined under pulmonary loads in the middle of the leaflet.

## Results

### G&R of planar tissues after biaxial extension

**Fig. 2:**
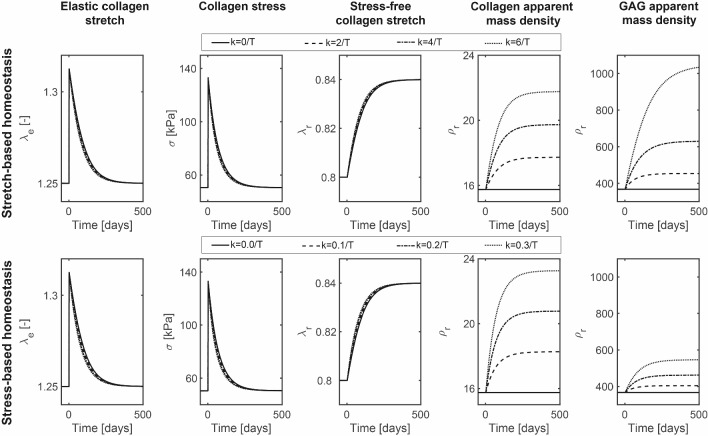
Tissue G&R after an equibiaxial 5% extension under the assumption of either stretch- or stress-based homeostasis simulated with various gain parameters

We first simulated the restoration of mechanical homeostasis after a 5% equibiaxial extension for both stretch- and stress-based homeostasis. In both cases, as a direct consequence of the instantaneous equibiaxial extension, the stretch and stress of the individual collagen fibers increased to values above their homeostatic levels (Fig. 2). As these overly stretched collagen fibers were degraded and replaced by collagen fibers that were stretched at their homeostatic level, there was a turnover-associated change in the stress-free stretch of the collagen fibers that, over time, returned the average fiber stretch and stress to its homeostatic level (Fig. 2). Variations of the gain parameters $k_{\lambda} ^i$ and $k_{\sigma} ^{i}$ over the ranges studied only had a mild effect on the rate of change in the stress-free configuration of collagen fibers and did not influence their ultimate values (Fig. 2).

In the period when the stretches (for stretch-based homeostasis) or stresses (for stress-based homeostasis) were above their homeostatic level, the mass deposition rate increased for simulations with $k_{\lambda} >0/T^i$ and $k_{\sigma }>0/T^i$, respectively, leading to higher apparent mass densities of collagen fibers and GAGs in both cases (Fig. 2). In contrast to the changes in the stress-free state of the collagen fibers, the increase in mass density highly depended on the additional out-of-homeostasis deposition rate ($k_{\lambda}$ and $k_{\sigma}$). In addition, apparent GAG densities increased substantially more under stretch homeostasis, whereas apparent collagen mass densities increased only slightly more in the situation of stress homeostasis (Fig. 2). This difference was related to the more linear stress–stretch relationship of GAGs compared to collagen and the corresponding higher gain parameters during stretch homeostasis. In summary, the G&R of a planar tissue in response to an equibiaxial extension is a combination of a mechano-mediated increase in tissue mass, where the change in composition depends on the assumption of either stretch- or stress-based homeostasis, and a turnover-associated increase in the stress-free stretch of the collagens fibers and GAGs.

**Fig. 3:**
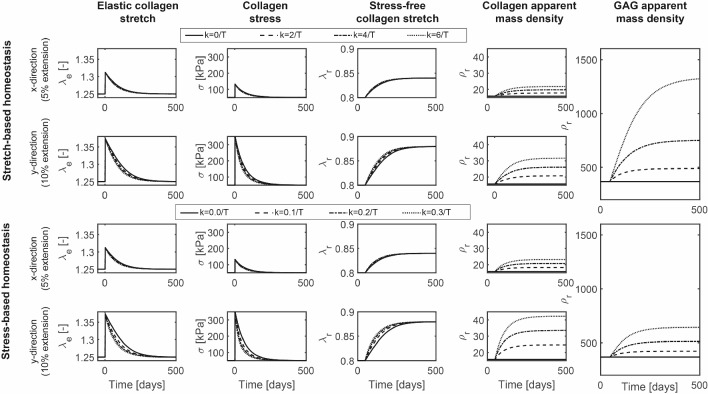
Tissue G&R after a 5%:10% biaxial extension under the assumption of either stretch- or stress-based homeostasis simulated with various gain parameters

**Fig. 4:**
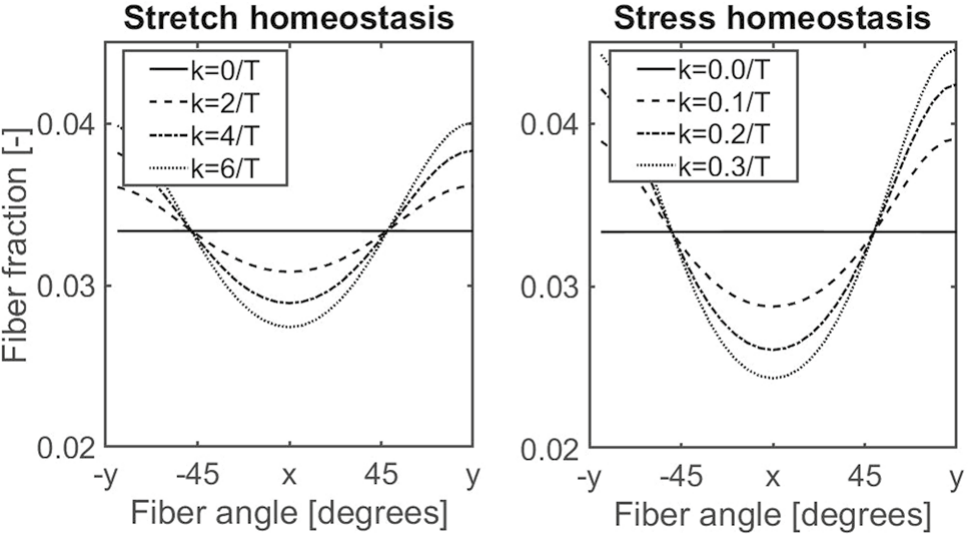
Final collagen fiber distribution after G&R under the assumption of stretch- (left) or stress-based (right) homeostasis

Next, we investigated the effect of loading anisotropy on tissue G&R by simulating a biaxial tensile test with different elongations in the *x*- and *y*-direction (5% and 10%, respectively). Again, the collagen fiber stretches and stresses increased (Fig. 3), with fibers oriented along the *y*-direction experiencing the highest levels. Similar to the case of equibiaxial extension, fiber stresses and stretches decreased over time through a turnover-mediated change in their stress-free configuration (Fig. 3). Since mass deposition is regulated by the stresses or stretches in the individual constituents, and in case of collagen also direction-dependent, the new homeostatic state featured an anisotropic collagen fiber distribution (Fig. 4), where the degree of anisotropy depended on the out-of-homeostasis deposition rates $k_{\lambda} ^i$ and $k_{\sigma} ^{i}$. The apparent GAG densities increased similarly to the case of the equibiaxial extension, although slightly higher values were reached due to a higher overall deformation compared to the previous equibiaxially loaded situation (Fig. 3). Taken together, the main difference between G&R of planar tissues under isotropic and anisotropic loading conditions is that anisotropic loading induces a natural anisotropy in the collagen fiber distribution.

### G&R after the Ross procedure with stress-based homeostasis

**Fig. 5:**
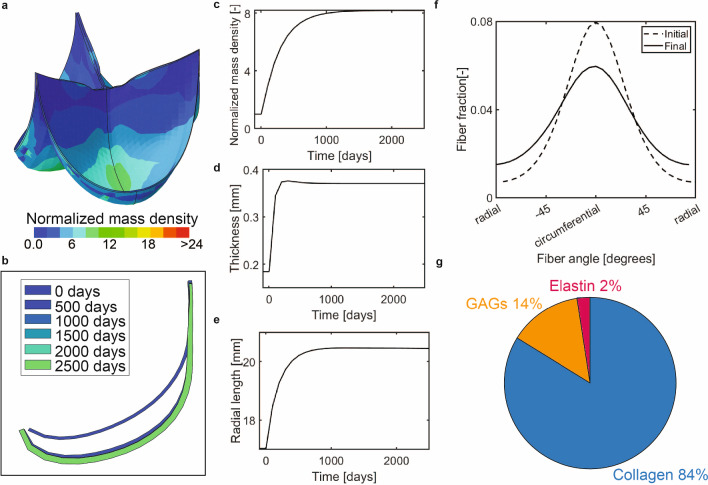
(**a**) Spatial distribution of the normalized mass increase under the assumption of stress homeostasis with $k_{\sigma} =0.3/T^{i}$. (**b**) Mid-section profile at different time points. (**c**) Normalized mass increase in the belly region over time. (**d**) Mid-belly thickness over time. (**e**) Radial arc length over time. (**f**) Homeostatic collagen fiber organization. (**g**) Homeostatic composition of the belly region

In the context of the Ross procedure, we first analyzed the G&R of pulmonary heart valves after an instantaneous increase in blood pressure toward aortic diastolic levels, without root dilatation, and under the assumption of stress homeostasis (with the highest out-of-homeostasis deposition rate $k_{\sigma} =0.3/T^{i}$). In general, the instantaneous increase in stress led to a substantial increase in apparent mass density and changes in valve geometry, collagen alignment, and tissue composition (Fig. 5). Importantly, a new homeostatic state was established within the simulated period of 2500 days.

The increase in mass density was predominantly in the belly region of the valve (Fig. 5A). This increase in mass (Fig. 5C) resulted in a relatively fast and substantial increase in leaflet thickness in the center of the leaflet (Fig. 5D), whereas the turnover-associated increase in leaflet length was smaller and appeared more gradually (Fig. 5E). These changes in leaflet thickness and length resulted in a deepening of the belly region and therefore dilation of the valve, which was most prominent in the period shortly after the increase in hemodynamic loading (Fig. 5B).

In terms of tissue composition, a significant increase in collagen volume fraction was predicted in the belly region (from 0.45 to 0.84, Fig. 5G). As collagen fibers carried the majority of the additional stress caused by the increase in hemodynamic loads, their deposition was increased more compared to the other ECM components. In contrast, the elastin volume fraction was greatly reduced (from 0.2 to 0.02) due to the large overall increase in volume while the absolute amount of elastin was stable over time. Differences in composition were present in different regions of the valve (Suppl. Fig. 2). Interestingly, there was a moderate reduction in the alignment of the collagen fibers due to G&R (Fig. 5F), although the fibers remained predominantly oriented in the circumferential direction. Since the initial main collagen direction was circumferential, the increase in load needed to be distributed over fewer fibers in the radial direction compared to the circumferential direction. As a consequence, the stress in the individual collagen fibers was increased more in the radial fibers (Suppl Fig. 3), which induced a larger increase in mass deposition in the radial direction compared to the circumferential direction, eventually leading to a reduced fiber alignment.

Overall, the increased loads after the Ross procedure invoked a G&R response that is mainly characterized by a thickening of the leaflet due to the increase in tissue mass and dilation due to the turnover of ECM components. Tissue composition changed considerably and became more collagen-dominated with a reduction in collagen alignment.

### G&R after the Ross procedure with stretch-based homeostasis

**Fig. 6:**
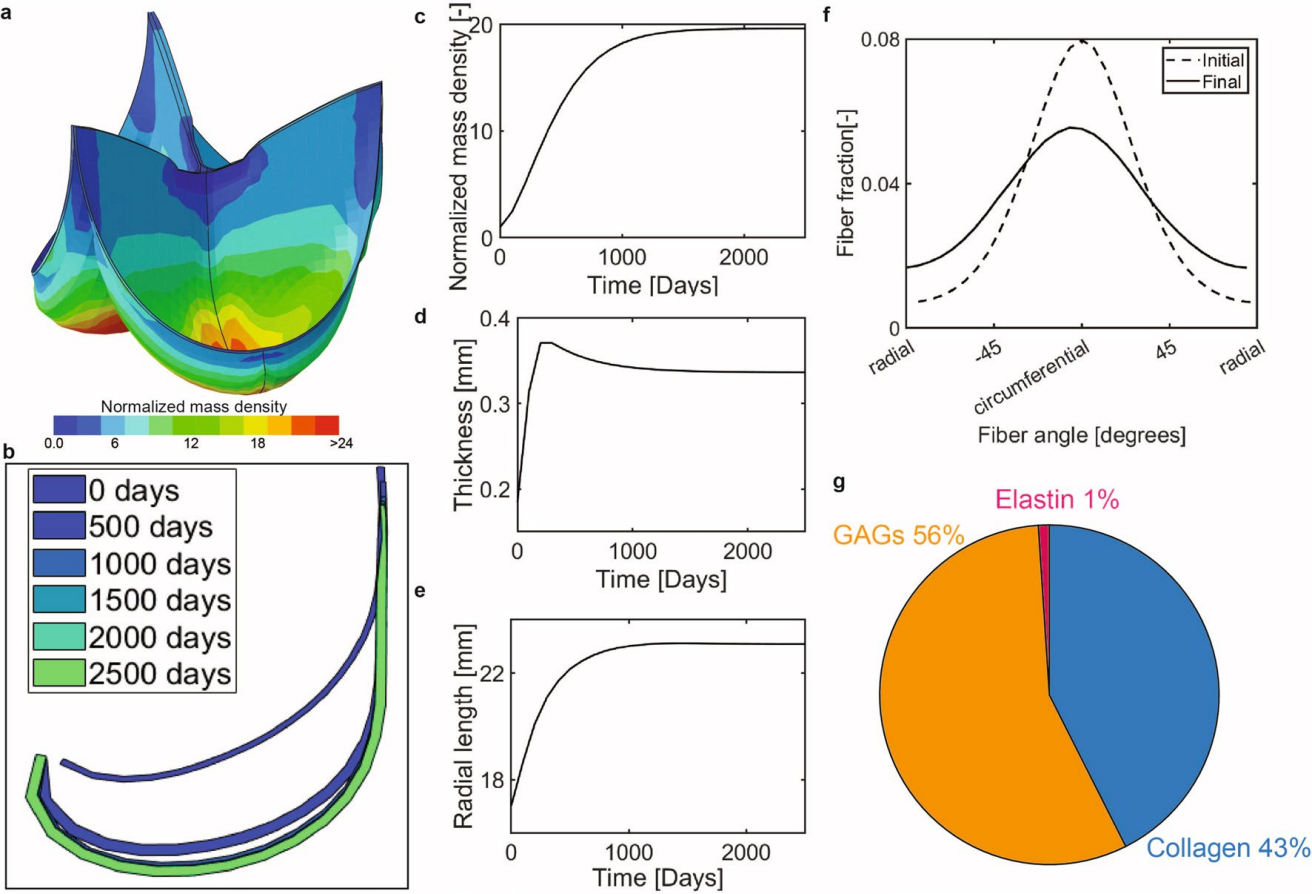
(**a**) Spatial distribution of the normalized mass increase under the assumption of stretch homeostasis with $k_{\sigma} =6/T^{i}$. (**b**) Mid-section profile at different time points. (**c**) Normalized mass increase in the belly region over time. (**d**) Mid-belly thickness over time. (**e**) Radial arc length over time. (**f**) Homeostatic collagen fiber organization. (**g**) Homeostatic composition of the belly region

Next, we simulated G&R after the Ross procedure under the assumption of stretch homeostasis (with $k_{\lambda} =6/T^i$). In general, the morphological changes of the valve due to preserving stretch homeostasis showed similar trends as those under stress homeostasis, and also in this case a new homeostatic state was obtained within the simulated period of 2500 days. However, although the spatial distribution of growth was similar (Fig. 6A), tissue mass increased substantially more in this situation compared to assuming stress homeostasis (Fig. 6A,C), due to the increased deposition of GAGs. Apart from this difference in the absolute amount of growth, and although slight differences in temporal profiles were present, the increase in mass again primarily increased leaflet thickness (Fig. 6D) and led to a larger, mainly turnover-mediated increase in leaflet length causing dilation of the valve (Fig. 6B,E).

The G&R of tissue constituents under the assumption of stretch homeostasis also resulted in a moderate decrease in collagen fiber anisotropy (Fig. 6F) and substantial changes in the tissue composition. Similar to stress homeostasis, the volume fraction of elastin was greatly reduced in the belly region (Fig. 6G) due to the production of collagen and GAGs. However, under the assumption of stretch homeostasis, GAG deposition increased much more compared to that of collagen, because the stimulus function for GAGs incorporates stretches in all three dimensions, leading to a GAG-dominated tissue composition (volume fraction of GAGs increased from 0.35 to 0.56). Local differences in composition were present as well (Suppl. Fig. 2).

Overall, given the diverse assumptions adopted, these results suggest that stress- and stretch-based homeostasis result in similar morphological changes and reductions in collagen alignment after the Ross procedure. The main difference is that a more GAG-dominated composition is obtained under stretch homeostasis, while a more collagen-dominated composition appears under the assumption of stress homeostasis.

### Effect of $k_{\lambda}$ and $k_{\sigma}$ on the G&R response after the Ross procedure

**Fig. 7:**
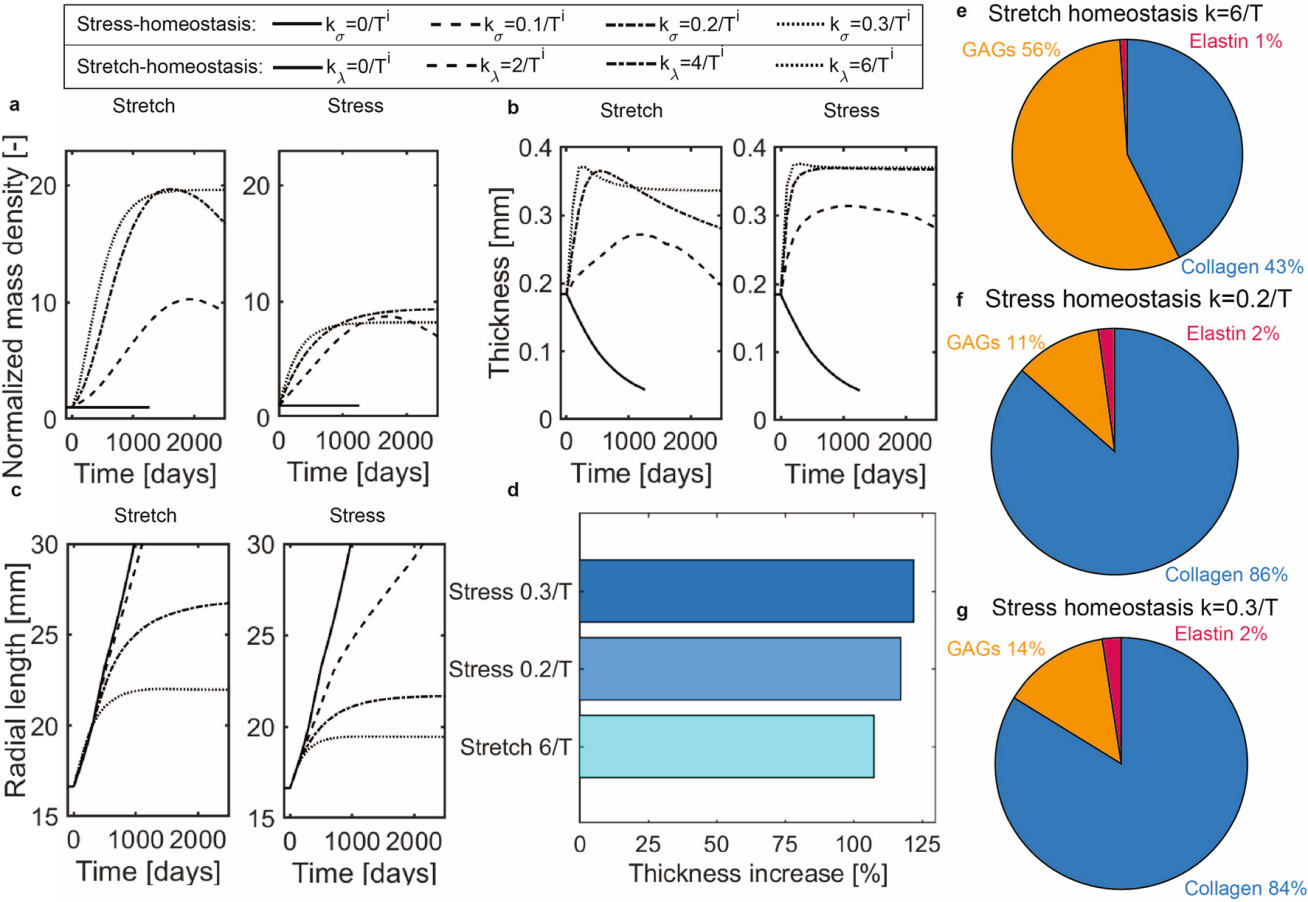
(**a**) Increase in belly mass over time. (**b**) The mid-belly thickness over time. (**c**) Radial arc length over time. (**d**) Thickness increase in the new homeostatic state. Composition in the new homeostatic state for: (**e**) stretch homeostasis ($k_{\lambda }=6/T^i$), (**f**) stress homeostasis ($k_{\sigma }=0.2/T^i$), and (**g**) ($k_{\sigma }=0.3/T^i$)

To investigate to what extent the strength of mechanobiological feedback between changes in mechanical loading and changes in ECM deposition rate affected the G&R response, we simulated four different values for $k_{\lambda}$ and $k_{\sigma}$ for stress- and stretch-based homeostasis (Fig. 7). These simulations revealed that an insufficient ability to adjust ECM deposition in response to a change in the mechanical environment results in excessive dilation and an inability to re-establish a homeostatic state.

Specifically, out of the eight different simulations, a stabilization of the normalized tissue mass in the belly region (Fig. 7A), the mid-belly thickness (Fig. 7B), and the leaflet length (Fig. 7C), indicating the re-establishment of homeostasis, was only predicted for three cases (stretch-homeostasis with $k_{\lambda} =6/T^i$ and stress-homeostasis with $k_{\sigma} =0.2/T^{i}$ and $k_{\sigma} =0.3/T^{i}$). In all other cases, the pulmonary valve demonstrated significant dilation and thinning, even in case of ECM growth, without re-establishing homeostasis. Furthermore, simulations in which the deposition of the constituents always balanced their degradation ($k_{\sigma} =0/T^{i}$ and $k_{\lambda }=0/T^i$) failed to achieve numerical convergence throughout the entire G&R period, most likely due to excessive dilation and associated thinning.

Changes in the out-of-homeostasis deposition rates also had an effect on how the increase in tissue mass translated into morphological changes of the pulmonary valve. For example, in the simulations where the total apparent mass density, and hence tissue volume, remained constant ($k_{\lambda} ^i=0$ and $k_{\sigma} ^{i}=0$), the turnover of ECM-constituents still resulted in a dilation-associated thinning of the leaflets (Fig. 7B). On the other hand, in the case of increased ECM production upon mechanical perturbations of ($k_{\lambda} ^i>0$ and $k_{\sigma} ^{i}>0$), the resulting increases in volume initially caused much larger increases in the thickness of the leaflets compared to the increases in leaflet length. Generally, leaflet thickness increased until a maximum value was reached, after which it decreased again to either stabilize at a new homeostatic thickness ($k_{\lambda} =6/T^i$ & $k_{\sigma} =0.3/T^{i}$) or continued decreasing without reaching homeostasis ($k_{\lambda} =2/T^i$, $k_{\lambda} =4/T^i$ & $k_{\sigma} =0.1/T^{1}$, Fig. 7B). Only one simulation (under stress homeostasis with $k_{\sigma} ^{i}=0.2/T^{i}$) did not follow these trends but instead stabilized at its maximum thickness. A comparison of the initial and final homeostatic thicknesses showed that leaflet thickness increased between 107 and 121% when homeostasis was re-established (Fig. 7D).

In addition to the changes in leaflet thickness, the geometry of the valve also changed due to increases in leaflet length. This lengthening initially occurred at similar rates for the different values of $k_{\sigma} ^{i}$ and $k_{\lambda} ^i$, but stabilization occurred at earlier times for higher values of $k_{\lambda} ^i$ and $k_{\sigma} ^{i}$ (Fig. 7C). These smaller leaflet lengths in cases of higher values of $k_{\lambda} ^i$ and $k_{\sigma} ^{i}$ therefore resulted in less dilatation of the pulmonary valve.

Finally, we compared tissue composition in the three newly established homeostatic states across different simulations. In all cases, elastin volume fraction decreased due to the increased production of collagen and GAGs (Fig. 7E-G). Additionally, for stress homeostasis, a considerable increase in the collagen volume fraction was predicted, with a negligible effect of the value for $k_{\sigma} ^{i}$. In contrast, when assuming stretch homeostasis, the increase in volume was primarily due to increased GAG production. Altogether, these results show that the predicted tissue composition primarily depends on the assumption of maintaining stress or stretch homeostasis, while the value of the out-of-homeostasis deposition rate $k_{\sigma} ^{i}$ in case of stress homeostasis seems to have a negligible effect on the final tissue composition.

### Effect of the rate of pressure increase on G&R after the Ross procedure

**Fig. 8:**
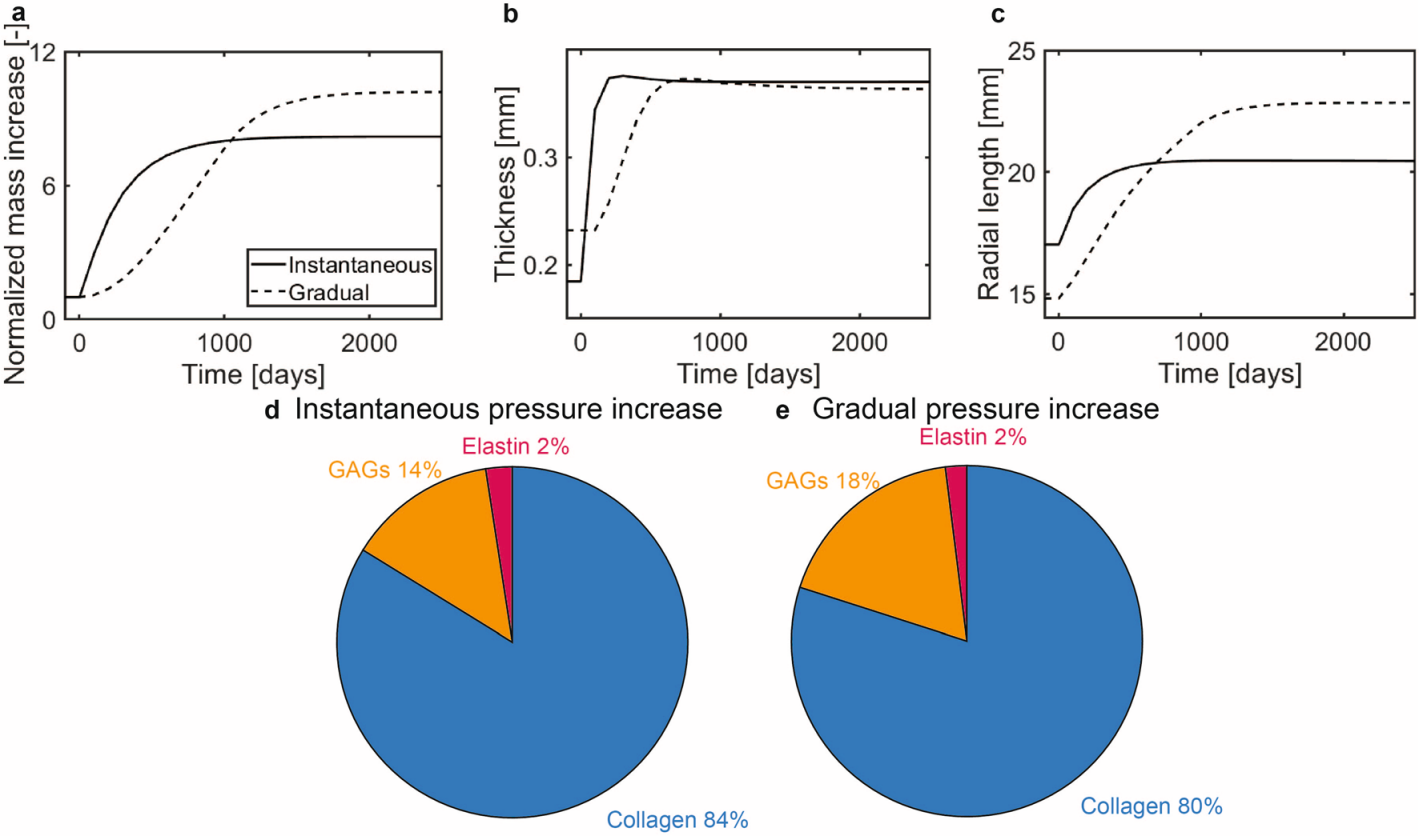
(**a**) Increase in belly mass over time due to an instantaneous or gradual increase in hemodynamic loading. (**b**) The mid-belly thickness over time. (**c**) Radial arc length over time. Composition in the new homeostatic state for: (**d**) an instantaneous increase in pressure, and (**e**) a gradual increase in pressure

When a more gradual increase in hemodynamic loads was simulated (with stress-based homeostasis and $k_{\sigma} =0.3/T^{i}$), a more gradual increase in normalized mass density in the belly region was observed as well (Fig. 8A). As G&R was already occurring during the increase in pressure, excessive constituent-level stresses were avoided, leading to a lower rate of mass deposition compared to the simulation with an instantaneous pressure increase. Nevertheless, due to the gradual increase in hemodynamic loading, the normalized mass density stabilized at a later time point compared to the situation of an instantaneous increase in pressure, ultimately resulting in a higher normalized mass density in the newly established equilibrium configuration.

As in the previous simulations, the mid-belly thickness increased over time, albeit more slowly compared to an instantaneous increase in pressure (Fig. 8B). Despite the lower rate of thickening, the final homeostatic thickness was independent of the rate of pressure increase. Interestingly, since more time was needed to establish homeostasis during a gradual increase in pressure, the ECM turnover-associated dilation in the radial direction continued for a longer period. As a consequence, the final leaflet length after re-establishing homeostasis was larger when the change in hemodynamic loading was applied more gradually (Fig. 8C). On the other hand, the final tissue composition was largely unaffected by the adapted time course of the pressure increase (Fig. 8E-F).

### Effect of dilation of the neo-aortic root on G&R after the Ross procedure

**Fig. 9:**
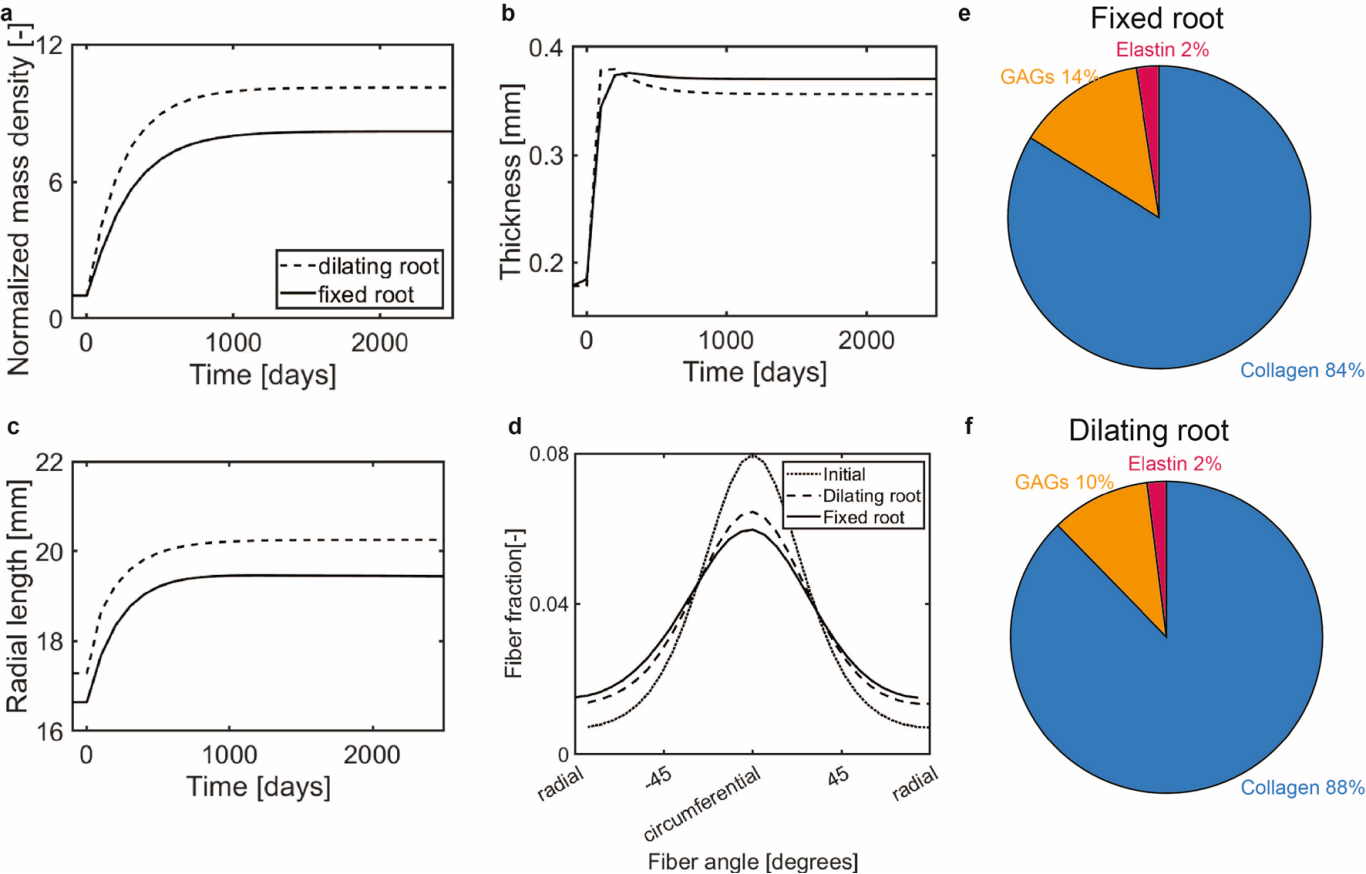
The effect of root dilation on G&R after the Ross procedure under the assumption of stress homeostasis with $k_{\sigma} =0.3/T^{i}$. **a** Normalized mass density of the belly region over time. **b** Leaflet thickness over time. **c** Radial arc length over time. **d** Collagen fiber distribution in the new homeostatic state. **e** Final homeostatic composition without root dilation. **f** Final homeostatic composition with root dilation

To study the effect of root dilation on heart valve G&R after the Ross procedure, we applied a 20% root dilation under the assumption of stress homeostasis with $k_{\sigma }=0.3/T^{i}$. Compared to the situation without root dilation, the additional stretches due to root dilation caused a larger increase in apparent mass density (Fig. 9A) due to the dilation-mediated increase in the GAG and collagen stresses. This additional increase in tissue mass primarily translated into a larger elongation of the valve in radial direction due to the larger in-plane stretches and was therefore accompanied by a slightly lower increase in leaflet thickness (Fig. 9B–C).

The dilation of the root also resulted in a reduced loss of anisotropy in the collagen fiber network within the leaflets (Fig. 9D). This can be explained by the fact that dilation of the root induced higher radial stresses in the leaflet leading to a larger increase in radially oriented collagen. On the other hand, the final tissue composition was hardly affected by the inclusion of root dilation (Fig. 9E–F). Finally, although an instantaneous increase in annular size may not necessarily represent the in vivo situations, we observed similar changes in the heart valves after a more gradual application of root dilation after the simulated Ross-procedure (Suppl. Fig. 4).

## Discussion

Living valve replacements have the potential to be superior to conventional replacements. This is partly due to their G&R ability that, in principle, enables living valves to adapt well to their new hemodynamic environment. As living valve replacements, Ross autografts generally perform well although they are susceptible to the development of valvular regurgitation (Takkenberg et al. 2009). To understand better how the G&R of autograft leaflets contributes to or prevents regurgitation, we developed a homogenized constrained mixture model of the pulmonary valve. This model extends the work of Cyron et al. (2016) by incorporating the distinct mechanical and microstructural properties of the pulmonary valve. These additions include GAGs as an ECM constituent that undergoes G&R, an elaborate description of the collagen network with multiple fiber directions for an improved description of the tissue anisotropy, and valve-specific mechanical properties of the ECM constituents. This model was then used to simulate the adaptation of pulmonary valves due to exposure to systemic blood pressures after the Ross procedure.

In all our investigated scenarios (stress- or stretch-based homeostasis, with or without blood pressure control, and with or without dilation of the neo-aortic root), our model consistently predicted the emergence of thicker and longer leaflets, where the majority of additional mass was deposited in the belly area of the valve, with a moderate decrease in the circumferential alignment of the collagen network and a substantial change in the tissue composition. The predicted thickening agreed well with observed increases in thickness in clinical studies, ranging between 27% and 300% (Mookhoek et al. 2010; Yacoub et al. 2020; Rabkin-Aikawa et al. 2004). Our finding that new tissue was mainly deposited in the belly area agrees with previous observations that the formation of an additional tissue layer was most evident in the leaflet belly; furthermore, the collagen in this additional layer was mainly radially oriented (Yacoub et al. 2020), which indicates a similar decrease in circumferential alignment in clinical studies and our simulations. The agreement between these observations and the predictions of our computational model demonstrates that heart valve adaptation after the Ross procedure can be captured well by G&R mechanisms aimed at restoring mechanical homeostasis.

The predicted changes in the autograft result from an extensive interplay between mechanical stimuli and mass deposition and turnover. In the initial stages after the Ross procedure, the changes in hemodynamic conditions led to higher elastic stretches and stresses in tissue constituents. As a consequence, mass production of collagen and GAGs increased, resulting in a larger tissue volume due to incompressibility. As demonstrated by Braeu et al. (2019), increases in tissue volume mainly manifest in the direction of the lowest stiffness, which in our case coincided with the thickness direction. This increase in thickness resulted in a higher structural stiffness of the leaflet, which in turn limited tissue level deformations, reducing the stretches and stresses of the tissue constituents. Together, these processes represent a negative feedback loop that, in principle, can restore constituent-level stretches (and corresponding stresses) to their homeostatic levels.

In addition to leaflet thickening, turnover-associated dilation was also observed in our simulations. In the leaflets, the mass that was deposited during the G&R response was subjected to lower elastic stretches than the already existing mass. As a consequence, the average stress-free configuration of the constituents changed, resulting in a turnover-associated lengthening of the tissue in directions where the elastic stretches exceeded homeostatic values. Therefore, the resulting dilation occurred mainly in the fibrous plane, perpendicular to the thickness direction. Since dilation does not affect the tissue mass, the resulting increase in leaflet length due to constituent turnover was associated with a decrease in leaflet thickness, which explains the observed decreases in leaflet thickness at the end of the G&R response. In summary, these two mechanisms, deposition-associated thickening and dilation-associated thinning, had counteracting effects, and homeostasis was only reached when the deposition-related increase in thickness was sufficient to compensate for the dilation-associated decrease in leaflet thickness. This phenomenon is well-known under the term of mechanobiological stability (Cyron and Humphrey 2014). After mechanical perturbations, biological tissues can enter a trajectory of unstable (potentially unlimited) deformation if mechanically regulated mass deposition does not compensate turnover-related dilatation. Mechanobiological instabilities are a class of instabilities that can occur in biological tissues subject to growth and remodeling in addition to classical purely mechanical instabilities (e.g., buckling). They have been hypothesized to be the governing mechanism also of aneurysms (Baek et al. 2005; Cyron et al. 2014).

Our simulations further revealed a consistent reduction in the circumferential alignment of the collagen network across all simulations, which was a consequence of a relatively greater increase in fiber stretches (or equivalent stresses) in the radial versus the circumferential direction. These predictions agree with experimental observations of the formation of a radially oriented layer of additional tissue after the Ross procedure (Yacoub et al. 2020). In addition, such changes in collagen orientation are not specific to the pulmonary autograft. A similar reduction in collagen alignment was observed in tricuspid valve leaflets after an increase in radial leaflet stress due to biventricular heart failure (Meador et al. 2020) and the mitral valve after an increase in stress due to annular dilation during pregnancy (Pierlot et al. 2014). This suggests that the reduction of circumferential collagen alignment after an increase in radial loading is a shared response of valve leaflets in different types of heart valves, which may be driven by the restoration of mechanical homeostasis at an individual fiber level.

This reduction in collagen alignment also highlights that it is not guaranteed that a living valve replacement will obtain the material properties of its native counterpart through G&R. The native aortic valve has a substantially stronger circumferential alignment of collagen fibers compared to the native pulmonary valve (Oomen et al. 2016; Soares et al. 2014). Yet, our model predicts that a pulmonary autograft in the aortic position will develop a more dispersed fiber organization, indicating that the autograft does not remodel toward an aortic-like valve but instead obtains a different equilibrium configuration. Our simulations suggest that this configuration allows proper closure, but further research is required to determine if such different tissue properties may negatively impact the valve’s long-term functionality or adaptive capacity.

In this study, we investigated three different aspects of heart valve G&R after the Ross procedure. First, potential differences between a G&R response based on stress- or stretch-homeostasis to analyze which hypothesis could best describe the adaptive response of heart valve leaflets. The main difference between these hypotheses was the predicted final tissue composition, which was more collagen-dominated under the assumption of stress homeostasis and more GAG-dominated under the assumption of stretch homeostasis. Although there is limited data on the composition of explanted autografts, changes have been reported to range from limited effects (Rabkin-Aikawa et al. 2004) to fibrosis of the ventricularis (Mookhoek et al. 2010) or the formation of a fibro-cellular overgrowth (Yacoub et al. 2020; Schoof et al. 2006). These experimental observations suggest that an increase in relative collagen content is more likely than an increase in GAG content, indicating that stress homeostasis may be a more likely target for mechano-mediated G&R than stretch homeostasis. Similar increases in collagen content have also been reported in mitral valve G&R during pregnancy (Pierlot et al. 2014), suggesting that the deposition of additional collagen to restore mechanical homeostasis is a shared response for different heart valves. Additional experimental evidence will be required to confirm this hypothesis, however.

Second, we studied the effect of a more gradual increase in blood pressure to simulate effects of blood pressure-lowering drugs, which are sometimes administered after the Ross procedure (El-Hamamsy et al. 2010) to avoid root-dilation. Our model predicted that this more gradual increase in blood pressure has limited effects on the composition or thickness of the leaflets, but the final leaflet length after G&R was larger compared to an instantaneous increase in blood pressure. These longer leaflets might indeed have a protective effect against developing regurgitation as a consequence of root dilation. Our simulations thus support the current hypothesis that these drugs may prevent the development of regurgitation and further suggest that lowering the blood pressure below physiological levels in combination with heavily restricted activity in the period after the procedure might be an interesting therapeutic strategy. Further studies are required to investigate the potential of this therapy.

Third, we considered the effect of root dilation on heart valve G&R after the Ross procedure. An instantaneous increase in radius was simulated as an extreme case, which mainly resulted in a larger leaflet length compared to the situation without root dilation due to additional turnover-mediated tissue dilation. This finding thus suggests that the leaflets can elongate as a consequence of root dilation, which may, at least to a certain extent, limit the development of regurgitation. Again, this response is not unique to the pulmonary valve; annular dilation in the mitral valve during pregnancy has also been shown to induce an increased leaflet size (Wells et al. 2012) to ensure that coaptation is maintained.

Notwithstanding the many insights gleaned, additional studies should include other effects. Although use of autologous tissue reduces inflammation, the surgical procedure itself will stimulate an inflammation-mediated reparative process that could influence valve G&R. Although many aspects of tissue G&R can be captured phenomenologically, coupled cell signaling to tissue-level models promise deeper understanding (cf. Irons et al. 2021) and should be pursued.

Limited valve-specific data were available to inform parameters related to the G&R response of Ross autografts. As a consequence, some parameters were adopted from studies that investigated G&R phenomena in other tissues. For this reason, and to increase the reliability of the conclusions that were drawn, we varied the parameters with the highest degree of uncertainty ($k_{\sigma}$ and $k_{\lambda}$). Despite the limited data available, the agreement between the observed changes in explants and the predictions of the proposed model suggests that the estimated parameters were adequately representative for the modeling of the Ross procedure.

Another limitation of the current study is the exclusion of annulus kinematics as a consequence of the variations in blood pressure during a cardiac cycle. Although this assumption is commonly used in heart valve simulations (Borazjani 2013, Oomen et al. 2016, Zhang et al. 2021, Rego et al. 2022), excluding annulus kinematics remains a limitation as these may induce additional stresses and stretches in the leaflets.

In conclusion, we developed a homogenized constrained mixture model for heart valve G&R and simulated possible G&R of autologous pulmonary valves after the Ross procedure. Based on the hypothesis that G&R aims to restore a homeostatic mechanical state, our model was able to capture changes in leaflet thickness, tissue composition, and anisotropy of the collagen network as observed in valve explants (Schoof et al. 2006; Yacoub et al. 2020; Rabkin-Aikawa et al. 2004; Mookhoek et al. 2010). In addition, our simulations demonstrated that re-establishing mechanical homeostasis requires leaflet thickening through additional mass deposition to overrule leaflet thinning due to ECM turnover-mediated tissue dilation. Finally, our model predicted that the collagen network of the autograft remodels toward a stable architecture with a reduced collagen fiber alignment, which is different from the collagen organization of a healthy aortic valve. In future, this model for heart valve G&R may also be used to study other instances of heart valve G&R, such as leaflet adaptation due to partial unloading after the implantation of a left ventricular assist device (van Rijswijk et al. 2017), or other valve-related interventions and pathologies. Additionally, this model may be extended in future to investigate how mechanical loading affects the transition of a scaffold toward a neovalve during heart valve tissue engineering and, as such, serve as a tool to rationally design and improve tissue-engineered heart valves.

## Supplementary Information

Below is the link to the electronic supplementary material.

Supplementary file 1 (pdf 1668 KB)

## Data Availability

All data and computational codes are available at 10.4121/0d75d445-0f1a-4e92-9b80-bec9afc10556.

## Derivation of equation 31

For GAGs, the second Piola–Kirchhoff stress was defined as

$$\begin{aligned} \textbf{S}^g=\frac{\mu _g\rho _R^g}{\varphi ^g}(\textbf{C}_r^{g,-1}-\textbf{C}^{-1}), \quad \quad \quad (25 \quad \text {repeated}) \end{aligned}$$

and by inserting equation 25 into equation 9 the GAG deposition second Piola–Kirchhoff stress was obtained to be:

37 $$\begin{aligned} \textbf{S}^g_{dep}=\mu ^g\frac{\rho _{R}^g}{\varphi ^g}\big (\textbf{C}_{r}^{g,-1}-\textbf{F}_r^{g,-T}\cdot \textbf{C}_{e,0}^{g,-1}\cdot \textbf{F}_r^{g,-1}\big ). \end{aligned}$$

Finally, the derivative of the GAG second Piola–Kirchhoff stress to the GAG elastic right Cauchy–Green tensor can be written as

38 $$\frac{{\partial {\mathbf{S}}^{g} }}{{\partial {\mathbf{C}}_{e}^{g} }} = \frac{1}{2}\mu \frac{{\rho _{R}^{g} }}{{\varphi ^{g} }}\left( {({\mathbf{F}}_{r}^{{ - 1}} \cdot {\mathbf{C}}_{e}^{{ - 1}} )\bar{ \otimes }({\mathbf{F}}_{r}^{{ - 1}} \cdot {\mathbf{C}}_{e}^{{ - 1}} ) + ({\mathbf{F}}_{r}^{{ - 1}} \cdot {\mathbf{C}}_{e}^{{ - 1}} )\underset{\raise0.3em\hbox{$\smash{\scriptscriptstyle-}$}}{ \otimes } ({\mathbf{F}}_{r}^{{ - 1}} \cdot {\mathbf{C}}_{e}^{{ - 1}} )} \right)$$

In equation 38, the upper and lower dyadic product are defined in Einstein notation as $\textbf{A}\overline{\otimes }\textbf{B}=A_{ik}B_{jl}e_ie_je_ke_l$ and $\textbf{A}\underline{\otimes }\textbf{B}=A_{il}B_{jk}e_ie_je_ke_l$, respectively. By combining equation 8 with equation 38, we obtain the following equation:

39 $$\begin{aligned} \left[ {\frac{{\rho _{R}^{{\dot{g}}} }}{{\rho _{R}^{g} }} + \frac{1}{{T^{g} }}} \right]\left[ {S^{g} - S_{{dep}}^{g} } \right] = & \left[ {\frac{1}{2}\mu \frac{{\rho _{R}^{g} }}{{\varphi ^{g} }}\left( {(F_{r}^{{g, - 1}} \cdot C_{e}^{{g, - 1}} )\bar{ \otimes }(F_{r}^{{g, - 1}} \cdot C_{e}^{{g, - 1}} )} \right.} \right. \\ & \left. {\left. {\left. { + (F_{r}^{{g, - 1}} \cdot C_{e}^{{g, - 1}} )\underset{\raise0.3em\hbox{$\smash{\scriptscriptstyle-}$}}{ \otimes } (F_{r}^{{g, - 1}} \cdot C_{e}^{{g, - 1}} )} \right):[2C_{e}^{g} \cdot \dot{F}_{r}^{g} \cdot F_{r}^{{g, - 1}} ]} \right)} \right] \\ \end{aligned}$$

The equation for $\mathbf {\dot{F}}_r^g$ can then be obtained by resolving the dot and double dot products in equation 39. To do this, Einstein notation was applied to write:

40 $$\begin{aligned} \left[ {\frac{{\rho _{R}^{{\dot{g}}} }}{{\rho _{R}^{g} }} + \frac{1}{{T^{g} }}} \right]\left[ {S_{{ab}}^{g} - S_{{ab,dep}}^{g} } \right] & = \left[ {\frac{1}{2}\mu \frac{{\rho _{R}^{g} }}{{\varphi ^{g} }}\left( {F_{{r,ai}}^{{g, - 1}} C_{{e,ic}}^{{g, - 1}} F_{{r,bj}}^{{g, - 1}} C_{{e,jd}}^{{g, - 1}} } \right.} \right. \\ & \quad \left. {\left. { + F_{{r,ak}}^{{g, - 1}} C_{{e,kd}}^{{g, - 1}} F_{{r,bl}}^{{g, - 1}} C_{{e,lc}}^{{g, - 1}} } \right)\left[ {2C_{{e,cy}}^{g} \dot{F}_{{r,yz}}^{g} F_{{r,zd}}^{{g, - 1}} } \right]} \right] \\ \end{aligned}$$

The double dot product can now be resolved as:

41 $$\begin{aligned} \left[ {\frac{{\rho _{R}^{{\dot{g}}} }}{{\rho _{R}^{g} }} + \frac{1}{{T^{g} }}} \right]\left[ {S_{{ab}}^{g} - S_{{ab,dep}}^{g} } \right] = & \frac{{\rho _{R}^{g} }}{{\varphi ^{g} }}\left( {F_{{r,ai}}^{{g, - 1}} C_{{e,ic}}^{{g, - 1}} C_{{e,cy}}^{g} \dot{F}_{{r,yz}}^{g} F_{{r,zd}}^{{g, - 1}} C_{{e,dj}}^{{g, - T}} F_{{r,jb}}^{{g, - T}} } \right. \\ & \left. { + F_{{r,ak}}^{{ - g,1}} C_{{e,kd}}^{{g, - 1}} F_{{r,dz}}^{{g, - T}} \dot{F}_{{r,zy}}^{{g,T}} C_{{e,yc}}^{{g,T}} C_{{e,cl}}^{{g, - T}} F_{{r,lb}}^{{g, - T}} } \right) \\ \end{aligned}$$

Since certain dot products result in the identity tensor, this expression may be shortened to:

42 $$\begin{aligned} \left[ {\frac{{\rho _{R}^{{\dot{g}}} }}{{\rho _{R}^{g} }} + \frac{1}{{T^{g} }}} \right]\left[ {S_{{ab}}^{g} - S_{{ab,dep}}^{g} } \right] = & \mu \frac{{\rho _{R}^{g} }}{{\varphi ^{g} }}\left( {F_{{r,ay}}^{{g, - 1}} \dot{F}_{{r,yz}}^{g} F_{{r,zd}}^{{g, - 1}} C_{{e,dj}}^{{g, - T}} F_{{r,jb}}^{{g, - T}} } \right. \\ & \left. { + F_{{r,ak}}^{{g, - 1}} C_{{e,kd}}^{{g, - 1}} F_{{r,dz}}^{{g, - T}} \dot{F}_{{r,zl}}^{{g,T}} F_{{r,lb}}^{{g, - T}} } \right) \\ \end{aligned}$$

Next, using $\textbf{F}_r^{g,-1}\cdot \textbf{C}_e^{g,-1}\cdot \textbf{F}_r^{g,-T}=\textbf{C}^{-1}$ we obtained:

43 $$\begin{aligned} \left[ {\frac{{\rho _{R}^{{\dot{g}}} }}{{\rho _{R}^{g} }} + \frac{1}{{T^{g} }}} \right]\left[ {S_{{ab}}^{g} - S_{{ab,dep}}^{g} } \right] = & \mu \frac{{\rho _{R}^{g} }}{{\varphi ^{g} }} \\ & \left( {F_{{r,ay}}^{{g, - 1}} \dot{F}_{{r,yz}}^{g} C_{{zb}}^{{ - T}} + C_{{az}}^{{ - 1}} \dot{F}_{{r,zl}}^{{g,T}} F_{{r,lb}}^{{g, - T}} } \right) \\ \end{aligned}$$

Finally, since $\textbf{C}^{-1},\textbf{F}_r^{g,-1}$ and $\dot{\textbf{F}}_r^g$ are symmetric we may write

44 $$\begin{aligned} \Big [\frac{\dot{\rho _R^g}}{\rho _R^g}+\frac{1}{T^g}\Big ] [\textbf{S}^g-\textbf{S}^g_{dep}]= 2\mu ^g\frac{\rho _R^g}{\varphi ^g} \textbf{F}_{r}^{g,-1}\cdot \dot{\textbf{F}}_{r}^g\cdot \textbf{C}^{-1}, \end{aligned}$$

which can be rewritten as:

$$\begin{aligned} \dot{\textbf{F}_r^g}=\frac{\varphi ^g}{2\mu ^g\rho _R^g} \big [\frac{\dot{\rho _R^g}}{\rho _R^g}+\frac{1}{T^g}\big ] \textbf{F}_r^g\cdot (\textbf{S}^g-\textbf{S}^g_{dep})\cdot \textbf{C} \quad \quad \quad (31\quad \text {repeated}) \end{aligned}$$
