# Supplementary material for: Computational analysis of heart valve growth and remodeling after the Ross procedure
Source: Biomech Model Mechanobiol. 2024 Sep 13;23(6):1889–907. doi: 10.1007/s10237-024-01874-y (PMC11554944; doi:10.1007/s10237-024-01874-y)
Supplement: Supplementary file 1 — (pdf 1668 KB) [file 10237_2024_1874_MOESM1_ESM.pdf]

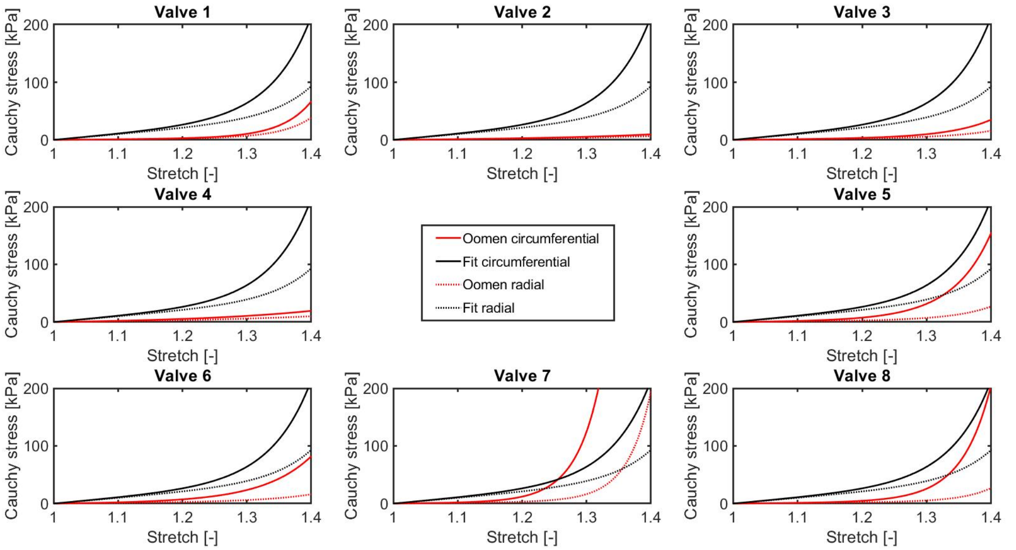

Fig. 1 Recreated stress-strain curves of Oomen et al (2016) and the fitted material model.

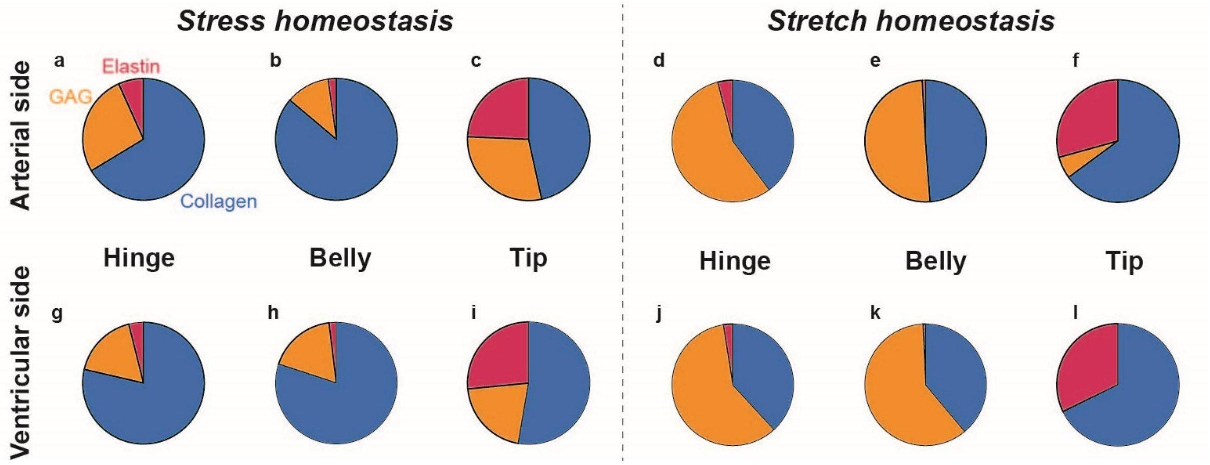

Fig. 2 Local composition of the autograft after G&R under stress (a-c, g-i) and stretch homeostasis (d-f, j-l) in the hinge (a,d,g,j), belly (b,e,h,k) and tip (c,f,i,l) regions at the top (a-f) and bottom g-l) of the leaflets.

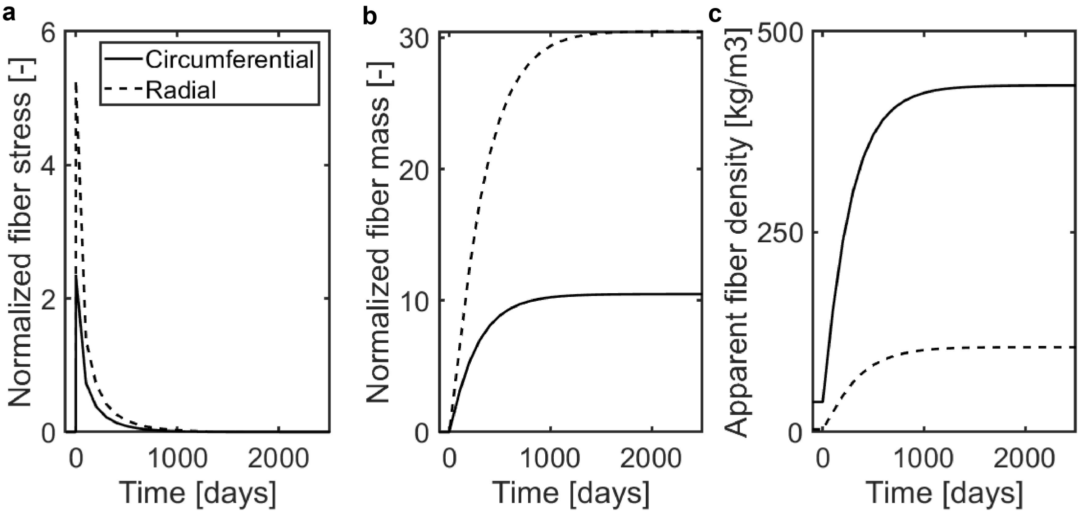

Fig. 3 **a** Stress in the circumferential and radial fiber normalized to the homeostatic fiber stress. **b** Density of the circumferential and radial fiber normalized to the initial fiber density. **c** Density of the circumferential and radial fiber.

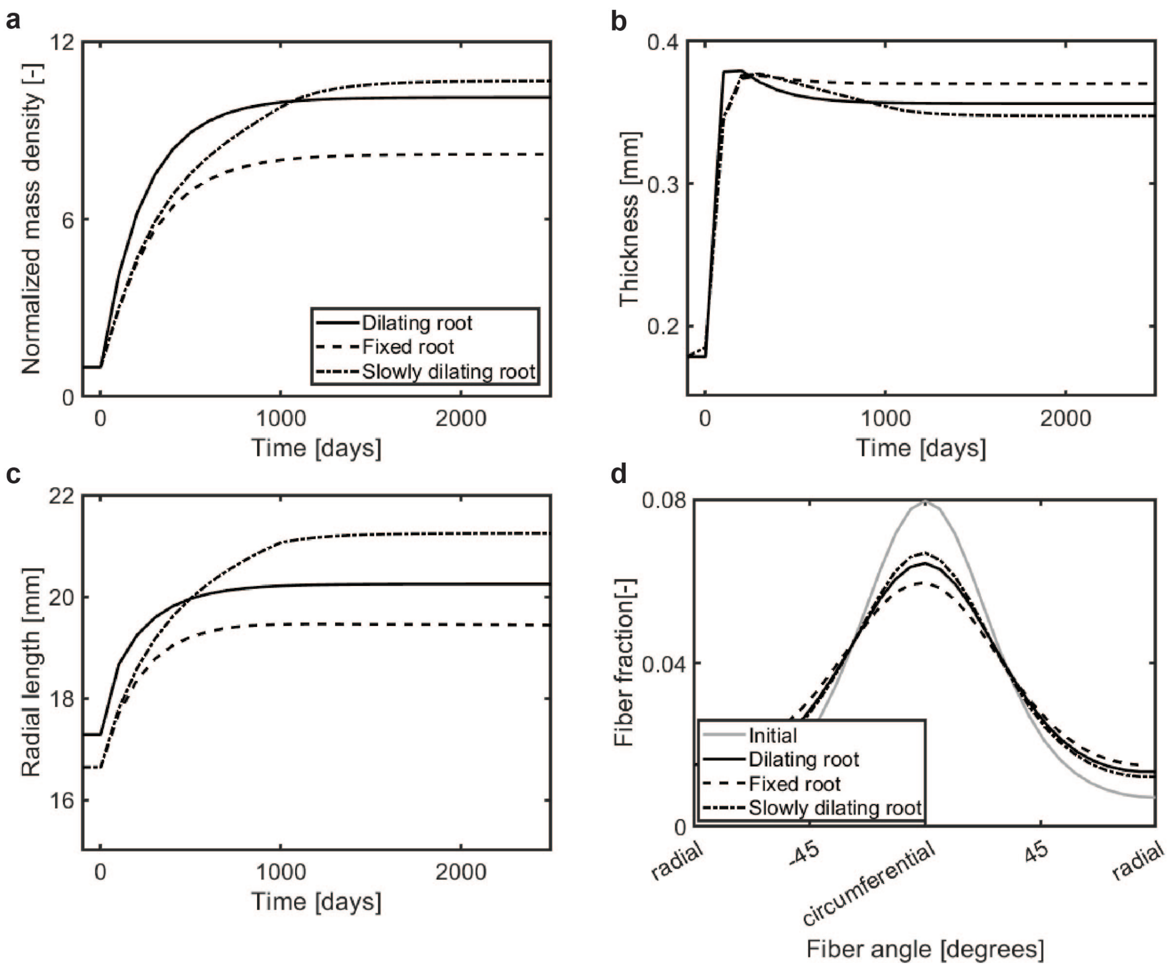

Fig. 4 The effect of root dilation rate on G&R after the Ross procedure under the assumption of stress homeostasis. **a** Normalized mass density of the belly region over time. **b** Leaflet thickness over time. **c** Radial arc length over time. **d** Collagen fiber distribution in the new homeostatic state. In the slowly dilating root, root dilation was prescribed as a 20% increase in radius in the first 1000 days of G&R.
